# Chinese Adaptation of the Bad Sobernheim Stress Questionnaire for Patients With Adolescent Idiopathic Scoliosis Under Brace Treatment

**DOI:** 10.1097/01.md.0000471261.17487.50

**Published:** 2015-08-28

In the article “Chinese Adaptation of the Bad Sobernheim Stress Questionnaire for Patients With Adolescent Idiopathic Scoliosis Under Brace Treatment”,^1^ which appeared in Volume 94, Issue 31 of *Medicine*, Dr. Qikai Huang's initials were incorrectly captured in the affiliations section. Dr. Huang is associated with the Department of Orthopedics at Shanghai Pudong Hospital. The article has since been corrected online.

## References

[1] Xu X Wang F Yang M Chinese adaptation of the bad Sobernheim stress questionnaire for patients with adolescent idiopathic scoliosis under brace treatment. *Medicine* 94 31:e1236. 26252283 10.1097/MD.0000000000001236 PMC4616594

